# Alisol B Alleviates Hepatocyte Lipid Accumulation and Lipotoxicity via Regulating RARα-PPARγ-CD36 Cascade and Attenuates Non-Alcoholic Steatohepatitis in Mice

**DOI:** 10.3390/nu14122411

**Published:** 2022-06-10

**Authors:** Zhuohui Zhao, Zhen-Tao Deng, Suling Huang, Mengmeng Ning, Ying Feng, Yu Shen, Qin-Shi Zhao, Ying Leng

**Affiliations:** 1 State Key Laboratory of Drug Research, Shanghai Institute of Materia Medica, Chinese Academy of Sciences, Shanghai 201203, China; 201628012342080@simm.ac.cn (Z.Z.); slhuang@simm.ac.cn (S.H.); ningmengmeng@aliyun.com (M.N.); yingfeng@simm.ac.cn (Y.F.); shenyu1125@simm.ac.cn (Y.S.); 2 University of Chinese Academy of Sciences, Beijing 100049, China; dengzhentaoa@mail.kib.ac.cn; 3 State Key Laboratory of Phytochemistry and Plant Resources in West China, Kunming Institute of Botany, Chinese Academy of Sciences, Kunming 650201, China

**Keywords:** Alisol B, non-alcoholic steatohepatitis, RARα, CD36

## Abstract

Non-alcoholic steatohepatitis (NASH) is a common chronic liver disease worldwide, with no effective therapies available. Discovering lead compounds from herb medicine might be a valuable strategy for the treatment of NASH. Here, we discovered Alisol B, a natural compound isolated from *Alisma orientalis* (*Sam.*), that attenuated hepatic steatosis, inflammation, and fibrosis in high-fat diet plus carbon tetrachloride (DIO+CCl_4_)-induced and choline-deficient and amino acid-defined (CDA)-diet-induced NASH mice. RNA-seq showed Alisol B significantly suppressed CD36 expression and regulated retinol metabolism in NASH mice. In mouse primary hepatocytes, Alisol B decreased palmitate-induced lipid accumulation and lipotoxicity, which were dependent on CD36 suppression. Further study revealed that Alisol B enhanced the gene expression of RARα with no direct RARα agonistic activity. The upregulation of RARα by Alisol B reduced HNF4α and PPARγ expression and further decreased CD36 expression. This effect was fully abrogated after RARα knockdown, suggesting Alisol B suppressed CD36 via regulating RARα-HNF4α-PPARγ cascade. Moreover, the hepatic gene expression of RARα was obviously decreased in murine NASH models, whereas Alisol B significantly increased RARα expression and decreased CD36 expression, along with the downregulation of HNF4α and PPARγ. Therefore, this study showed the unrecognized therapeutic effects of Alisol B against NASH with a novel mechanism by regulating RARα-PPARγ-CD36 cascade and highlighted Alisol B as a promising lead compound for the treatment of NASH.

## 1. Introduction

Non-alcoholic fatty liver disease (NAFLD) is a common metabolic disorder with a broad spectrum ranging from simple steatosis (NAFL) to non-alcoholic steatohepatitis (NASH), fibrosis, and cirrhosis, with a prevalence of approximately 25% worldwide [1]. However, the pathogenesis of NAFLD remains incompletely understood. The “two hit hypothesis” or “multiple hit hypothesis” states that hepatic lipid accumulation together with other pathological factors such as pro-inflammatory response, lipid peroxidation, and oxidative stress promote simple fatty liver to NASH [2,3]. As a progressive form of NAFLD, NASH may deteriorate further to cirrhosis and hepatocellular carcinoma [4]. However, no effective therapies have been clinically approved for NASH until now.

Hepatic fat accumulation results from a combination of increased circulating free fatty acid (FFA) uptake, increased de novo lipogenesis, and decreased hepatic β-oxidation [5]. Uptake of circulating lipids is one of the major metabolic processes keeping hepatic lipid homeostasis. Known as a transporter controlling FFA uptake, cluster of differentiation 36 (CD36) has been demonstrated to be closely associated with the progression of NAFLD. A clinical study revealed that an excessive hepatic fatty acid influx existed in NAFLD patients [6], along with the abnormal increased expression of hepatic CD36 [7]. Liver-specific disruption of CD36 reduced liver lipid content and improved insulin sensitivity in HFD-fed mice [8]. Although several transcriptional factors such as peroxisome-proliferator-activated receptors (PPARs), liver X receptor (LXR), pregnane X receptor (PXR) [9], aryl hydrocarbon receptor (AHR) [10], and endogenous molecules such as fatty acids and ox-LDL [11] were reported to regulate CD36 expression, the molecular mechanisms upon modulating CD36 expression remain incompletely understood. Moreover, although contributing to hepatic lipid homeostasis, CD36′s role on lipotoxicity and its involvement in NASH procession have not been fully investigated.

Retinoic acid receptors (RARs), composed of three subtypes (RARα, RARβ, and RARγ), belong to the steroid/thyroid superfamily of nuclear hormone receptors. RARα is expressed in most tissues, whereas RARβ is mainly expressed in central nervous system [12] and RARγ is mainly expressed in skin, epithelium, and cartilage [13]. RARs are activated by the biologically active metabolite of retinol such as all-trans retinoic acid (atRA) and 9-cis retinoic acid [14], and form heterodimers with retinoid X receptor (RXR) to regulate gene transcription and signal transduction pathways. As the major subtype among RARs, RARα is reported tightly associated with obesity, diabetes, and other metabolic diseases. Early studies showed that RARα dominant negative form caused steatohepatitis in transgenic mice [15]. Overexpression of RARα and dietary administration of atRA [16] or RARα agonist [17] alleviated hepatic lipid accumulation. Despite a few studies discovering diverse mechanisms, such as reducing fatty acid-dependent PTEN phosphorylation [18], improving leptin-dependent insulin sensitivity [17], or regulating HNF4α-mediated PPARγ expression [19], the precise molecular mechanisms of RARα on hepatic steatosis have not been fully elucidated. Moreover, as the key regulator maintaining lipid homeostasis, whether CD36 is involved in RARα’s regulation on hepatic lipid metabolism has not been reported so far. In addition, although atRA and synthetic RAR agonists were reported to alleviate NAFLD in preclinical studies, little research has focused on the therapeutic potential of increasing RARα expression and regulating RARα-mediated transcriptional cascade for the treatment of NASH.

*Alisma orientalis* (*Sam.*) is a well-known medicinal plant widely used in traditional and modern medicine, and its chemical constituents are identified more than 120 compounds including guaiane-type sesquiterpenes, protostane-type triterpenes, and guaiane-type and kaurane-type diterpenes [20]. *Alisma orientalis* (*Sam.*) has been proposed to have therapeutic effects on metabolic syndrome [21] such as insulin resistance, hyperlipidemia, obesity, and NAFLD [22]. Early studies revealed that the active constituents of *Alisma orientalis* (*Sam.*) such as Alisol A 24-acetate and Alisol B 23-acetate had anti-NAFLD activity, and the molecular mechanisms might be partly linked with AMPK [23], adiponectin [24], or activation of Farnesoid X receptor (FXR) [25,26,27]. However, as a major protostane triterpene isolated from *Alisma orientalis* (*Sam.*), Alisol B was only reported to have anti-hyperlipidemia [28] activity, and its effects against NASH have not been studied so far. Hence, in the present study, the therapeutic effects of Alisol B against NASH were evaluated in two widely used murine NASH models. Moreover, the molecular mechanisms of Alisol B on hepatic lipid accumulation and lipotoxicity via regulating RARα-PPARγ-CD36 cascade were explored.

## 2. Materials and Methods

### 2.1. Extraction of Alisol B from Alisma orientalis (Sam.)

Alisol B (purity > 97%) was extracted and purified, and the structure was confirmed by comparing the ^1^H, ^13^C NMR, and MS data with reported data [29]. Dried rhizomes of Alisma orientale were extracted three times with 95% ethanol and then concentrated under vacuum to yield a crude extract, which was suspended in H_2_O followed by partition with AcOEt. The AcOEt extract was subjected to medium-pressure liquid chromatography (MPLC) over MCI gel with methanol (40, 45, 50, 55, 60, 65, 70, 75, and 80%) to afford six fractions (fraction A–F). Alisol B was isolated from fraction D by silica gel CC using petroleum ether/AcOEt 8:2 as eluent.

### 2.2. Animal Experiments

Male C57BL/6J mice, purchased from Shanghai Ling Chang Laboratory Animal Care Co Ltd. (Shanghai, China), were housed in a temperature- and humidity-controlled room (22–24 °C, 50–60% relative humidity) under a 12 h light–dark cycle with free access to water and food. The animal experiments were approved by the Institute Animal Care and Utilization Committee (IACUC) of Shanghai Institute of Materia Medica (SIMM), Chinese Academy of Sciences (CAS) (no. 2019-01-LY-81).

For the DIO+CCl_4_-induced murine NASH model, C57BL/6J mice (4–5 weeks old) were fed with chow or high-fat diet (60% calories as fat, Cat#D12492i, Research Diet, New Brunswick, NJ, USA) for 8 weeks and assigned into five groups according to their body weight (*n* = 8). Then, the mice were orally administered with Alisol B, obeticholic acid (OCA, purity > 98%, Cat#HY-12222, MedChemExpress, Monmouth Junction, NJ, USA), or vehicle (0.25% CMC-Na, *w*/*v*) once daily, and intraperitoneally injected with CCl_4_ (0.05 mL/kg, Aladdin, Shanghai, China) or vehicle (olive oil) twice weekly for further 5 weeks. Experimental groups were as follows: normal control group (fed with chow diet, treated with 0.25% CMC-Na and olive oil), DIO group (fed with high-fat diet, treated with 0.25% CMC-Na and olive oil), DIO+CCl_4_ group (fed with high-fat diet, treated with 0.25% CMC-Na and 0.05 mL/kg CCl_4_), DIO+CCl_4_+Alisol B group (fed with high-fat diet, treated with 100 mg/kg Alisol B and 0.05 mL/kg CCl_4_), and DIO+CCl_4_+OCA group (fed with high-fat diet, treated with 30 mg/kg OCA and 0.05 mL/kg CCl_4_). At the end of the experiment, mice were fasted for 6 h and sacrificed. Blood samples and liver tissues were collected for subsequent analysis.

For CDA-diet-induced murine NASH model, C57BL/6J mice (7–8 weeks old) were fed with chow or CDA diet (Cat#A06071302, Sysebio, Changzhou, China) for 8 weeks, and divided into three groups (*n* = 8) on the basis of body weight, serum alanine aminotransferase (ALT), and aspartate aminotransferase (AST). Then, the mice were orally administered with Alisol B or vehicle (0.25% CMC-Na, *w*/*v*) once daily for further 8 weeks. Experimental groups were as follows: normal control group (fed with chow diet, treated with 0.25% CMC-Na), CDA group (fed with CDA diet, treated with 0.25% CMC-Na), and CDA+Alisol B group (fed with CDA diet, treated with 100 mg/kg Alisol B). At the end of the experiment, the mice were fasted for 6 h and sacrificed. Blood samples and liver tissues were collected for subsequent analysis.

### 2.3. Biochemical Assays

Serum ALT and AST levels were measured by automatic biochemistry analyzer (HITACHI, Tokyo, Japan) with the relative commercial assay kits (Shino-test Corporation, Tokyo, Japan). Hepatic malondialdehyde (MDA) and glutathione (GSH) levels were measured by commercial kits from Beyotime Biotechnology Institute (Shanghai, China) and normalized by tissue weight. Hepatic and cellular triglyceride (TG) levels were detected according to the manufacturer’s protocols (DongOu Biotech, Wenzhou, China) and normalized by tissue weight and protein content, respectively.

### 2.4. Histological Analysis and Definition of Scoring System

Liver samples were fixed in 4% paraformaldehyde, embedded in paraffin, and stained with hematoxylin–eosin (H&E) or Sirius Red. H&E staining was used for assessment of liver histology, and Sirius Red staining was used for assessment of liver fibrosis. The images were captured from five randomly selected fields in each liver section, and the NAFLD activity score was evaluated blindly according to the NAS grading score system by the American Association for the Study of Liver Disease (ASSLD) [30] at 200× magnification. Total NAS score was calculated as a sum of the scores for steatosis (including macro- and micro-vesicular steatosis separately), ballooning, and inflammation (scored by analyzing the amount of inflammatory foci/field). Collagen deposition was calculated using Image Pro Plus under the whole slice scan by Nano Zoomer 2.0 HT (Hamamatsu Photonics, Hamamatsu, Japan).

### 2.5. RNA-Seq Analysis

Total RNA was extracted from liver tissues of vehicle and Alisol B-treated DIO+CCl_4_-induced mice. Transcriptome sequencing experiments were performed by Majorbio Bio-Pharm Technology (Shanghai, China). The Deseq2 R package was used for differential gene expression analysis. The significant different genes were selected by |log2FC| ≥ 1 and *p*-value < 0.01. Kyoto Encyclopedia of Genes and Genomes (KEGG) analyses were performed to identify differentially expressed genes that were significantly enriched in the pathways.

### 2.6. Cell Culture and Treatment

Mouse primary hepatocytes were isolated from male C57BL/6J mice (8–12 weeks old) using a Seglen’s two-step method, as described [31], and cultured in minimum essential medium (MEM) supplemented with 10% (*v*/*v*) fetal bovine serum (FBS, Cat#10099, Gibco, Grand Island, NY, USA), 100 nM insulin (Sigma-Aldrich, St. Louis, MO, USA), and 10 nM dexamethasone (Sigma-Aldrich, St. Louis, MO, USA). The human hepatoma cell line Huh7 and human embryonic kidney cell line HEK293, purchased from Cell bank of Shanghai Institutes of Biological Sciences, Chinese Academy of Sciences (Shanghai, China), were cultured in Dulbecco’s modified Eagle’s medium (DMEM) containing 10% (*v*/*v*) FBS. All cell cultures were maintained under a humidified environment at 37 °C with 5% CO_2_.

### 2.7. Oil Red O Staining and Fatty Acid Uptake

Mouse primary hepatocytes were exposed to 0.2 mM palmitate (PA) with or without Alisol B for 48 h. After being fixed in 4% paraformaldehyde for 30 min, cells were stained with Oil Red O (Cat#71029781, Sinopharm Chemical Reagent Co., Shanghai, China) working solution for 20 min and subsequently stained with hematoxylin solution for 3 min. The respective images of Oil Red O staining were captured with an Olympus IX73 microscope at 200× magnification. To evaluate hepatic fatty acid uptake, mouse primary hepatocytes were treated with or without Alisol B for 48 h and then incubated with 100 nM BODIPY-C16 (Cat#3821, Thermo Fisher Scientific, Waltham, MA, USA) for 5 min. After being fixed with 4% paraformaldehyde for 30 min, the hepatocytes were incubated with DAPI to stain nuclei for 3 min, and the images of BODIPY-C16 fluorescence were captured at 600× magnification with an Olympus FV1000-SIM microscope.

### 2.8. Reactive Oxygen Species (ROS) Determination

DCFH-DA (Cat#287810, Sigma, Shanghai, China) was used to detect intracellular ROS in mouse primary hepatocytes. Mouse primary hepatocytes were seeded at a density of 2 × 10^5^ cells/mL and incubated overnight. The cells were treated with 0.2 mM PA in the presence or absence of Alisol B for 48 h, and DCFH-DA (20 μM) was added in the last 15 min. The fluorescence was detected at 499/525 nm by the microtiter plate reader.

### 2.9. RNA Interference

CD36, RARα, and FXR small interfering RNA (siRNA) were obtained from Gene Pharma (Shanghai, China). Mouse primary hepatocytes were transfected with appropriate siRNA or negative control siRNA for 24 h using Lipofectamine™ 2000 (Thermo Fisher Scientific, MA, USA) according to the manufacturer’s instructions.

### 2.10. Transient Plasmid Transfection

RARα plasmid (EX-Mm21043-M02) was purchased from GeneCopoeia (Shanghai, China). Mouse primary hepatocytes were transfected with plasmid or vector control for 24 h using Lipofectamine™ 2000 according to the manufacturer’s instructions.

### 2.11. Luciferase Reporter Activity Assay

Luciferase reporter activity assay was carried out on the basis of the reported approach [32]. Huh7 cells and HEK293 cells were seeded at a density of 2 × 10^5^ cells/mL and cultured overnight. Cells were transfected with hFXR or hRARα receptor expression plasmid (GeneCopoeia, Shanghai, China) and luciferase reporter plasmid (Genomeditech, Shanghai, China) using Lipofectamine™ 2000 for 6 h, and then were treated with Alisol B for 18 h. Luciferase reporter activity was measured by the Steady-Glo^®^ Luciferase Assay System (Promega, Madison, WI, USA) according to the manufacturer’s instructions.

### 2.12. Quantitative RT-PCR

RNA was isolated using Trizol reagent (Thermo Fisher Scientific, MA, USA) according to the manufacturer’s instructions. Gene expression studies were performed using Prime-Script RT Reagent Kit with gDNA Eraser and SYBR Premix Ex Taq kit (Takara Biomedical Technology, Dalian, China) and detected by ABI Prism VIIA7 Sequence Detection System. GAPDH mRNA was used as an endogenous control to normalize expression levels, and all primer sequences used in this study are included in Table 1.

### 2.13. Western Blot Analysis

Western blot analyses were performed according to previous protocols [29]. Briefly, proteins were extracted from the cells or liver tissues by lysis buffer. Protein concentrations were determined, and the equal amounts of lysates were loaded, separated by SDS-PAGE gels, and transferred to PVDF membranes. The membranes were blocked and incubated with primary antibodies overnight at 4 °C followed by an incubation with HRP-conjugated secondary antibody. Signals were detected by an ECL system (GE Healthcare, Buckinghamshire, UK) and band intensities were quantified by densitometry using the Quality One software (Bio-Rad, Hercules, CA, USA). The primary antibodies against α-SMA (Cat#19245), CD36 (Cat#14347), P-JNK1/2 (Cat#4668), JNK1/2 (Cat#9252), P-NF-κB (Cat#3033), NF-κB (Cat#8242), and GAPDH (Cat#5174) were purchased from Cell Signaling Technology (Danvers, MA, USA), and the primary antibody against Col-1a1 (Cat#A1352) was purchased from ABclonal Technology (Wuhan, China).

### 2.14. Statistical Analysis

Analyses and graphs presented were performed with GraphPad Prism 8 (GraphPad Software, La Jolla, CA, USA). All results were expressed as mean values ± SD. Statistical significance was analyzed by two-tailed unpaired t-test between two groups. The comparison of multiple groups was performed by one-way ANOVA followed by Tukey’s post hoc tests. Statistically significance was defined at *p* value < 0.05.

## 3. Results

### 3.1. Alisol B ameliorated NASH in a DIO+CCl_4_-Induced Murine Model

**Figure 1:**
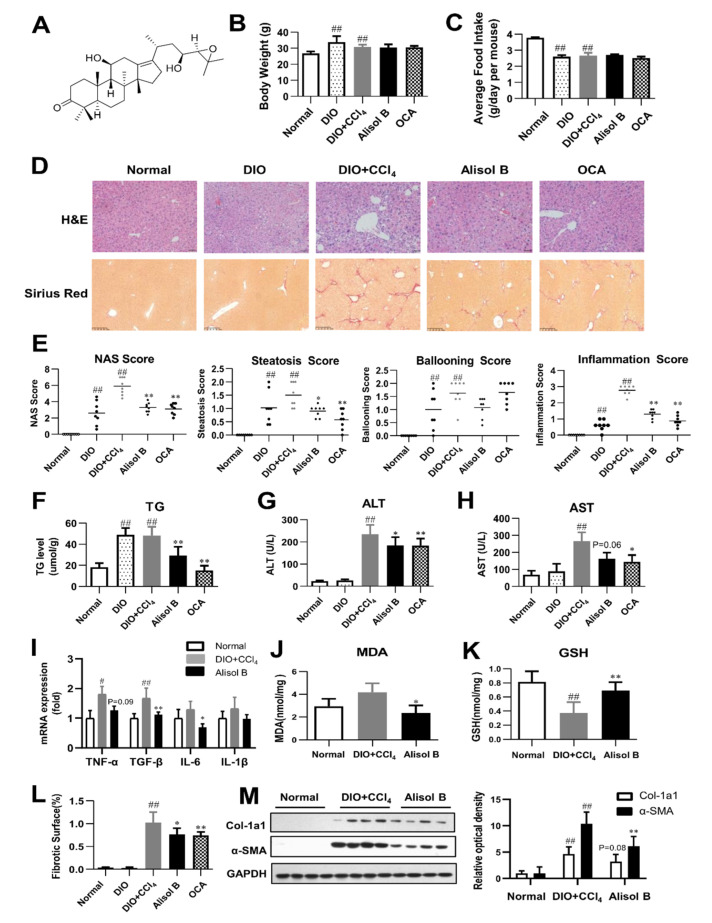
Alisol B ameliorated NASH in a DIO+CCl_4_-induced murine model. (**A**) The chemical structure of Alisol B. The therapeutic effects of Alisol B (100 mg/kg, once daily, p.o.) were evaluated in DIO+CCl_4_-induced mice as described in the Materials and Methods. (**B**) Body weight and (**C**) average food intake were measured. (**D**) H&E staining (200× magnification) and Sirius Red staining (100× magnification) were performed. (**E**) NAS score, steatosis score, ballooning score, and inflammation score were quantified. (**F**) Liver TG, (**G**) serum ALT and (**H**) AST, (**I**) hepatic mRNA levels of inflammatory cytokines, (**J**) hepatic MDA, and (**K**) GSH levels were measured. (**L**) Percentage of collagen deposition was calculated. (**M**) Western blot analysis of hepatic α-SMA and Col-1a1 expression was conducted and quantified. Data are presented as the mean ± SD, *n* = 8. # *p* < 0.05, ## *p* < 0.01 compared with the normal control group; * *p* < 0.05, ** *p* < 0.01 compared with the DIO+CCl_4_ group.

The therapeutic effects of Alisol B were first evaluated on a DIO+CCl_4_-induced model. After a 5-week Alisol B treatment, no significant differences were observed on body weight and average food intake (Figure 1B,C). The histological examination by H&E staining and Sirius Red staining revealed that the mice treated with high-fat diet plus CCl_4_ developed severe phenotypes of NASH. Alisol B significantly attenuated hepatic steatosis, ballooning, inflammation, and fibrosis compared with the DIO+CCl_4_ group (Figure 1D). Consistent with the improving pathological manifestations, obvious decreases in NAS, steatosis, and inflammation score (Figure 1E) were observed in the Alisol B-treated group. The hepatic TG content was also significantly decreased by 39.5% upon Alisol B treatment (Figure 1F). In addition, administration of Alisol B reduced serum ALT and AST levels (Figure 1G,H), indicating an attenuation of liver injury. This improvement was accompanied with the decreased transcript levels of inflammatory cytokines such as TGF-β, TNF-α, IL-6 (Figure 1I), and protective effects on lipid peroxidation indicated by lowered hepatic MDA level and increased GSH level (Figure 1J,K). The quantitative morphometric analysis of Sirius Red staining revealed that Alisol B treatment significantly decreased the percentage of collagen deposition (Figure 1L). Meanwhile, the protein levels of α-SMA and Col-1a1, two fibrotic markers upregulated in the DIO+CCl_4_ group, were also markedly suppressed by the treatment of Alisol B (Figure 1M).

### 3.2. Alisol B Ameliorated NASH in a CDA Diet-Induced Murine Model

**Figure 2:**
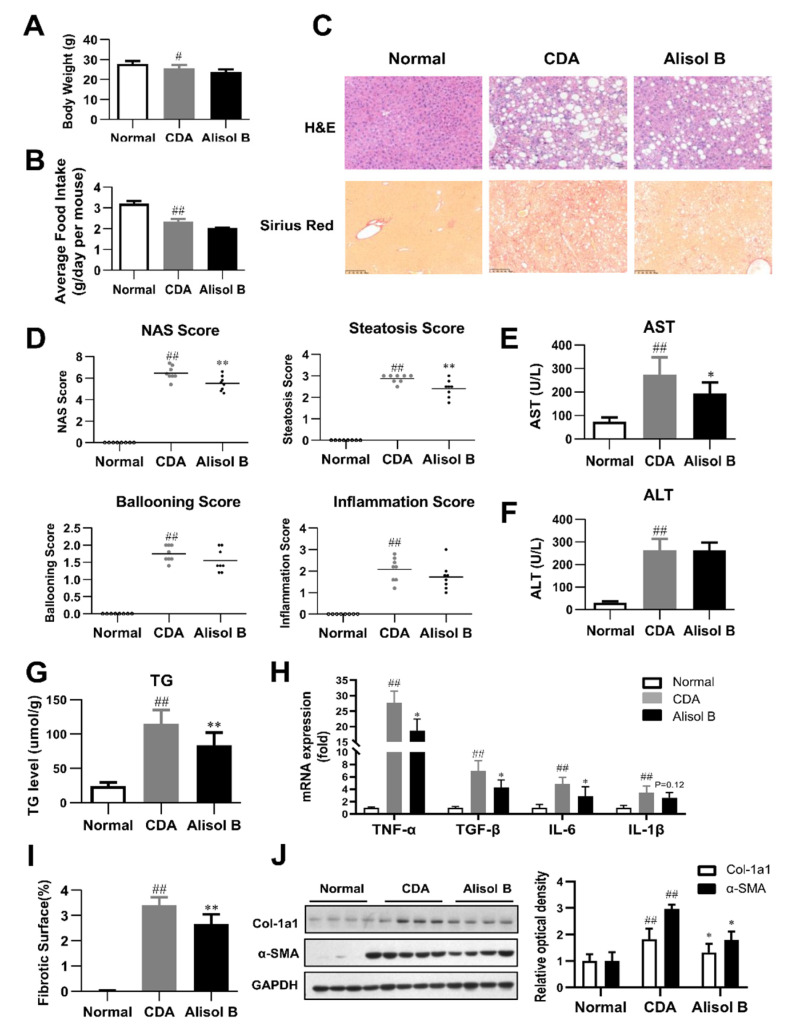
Alisol B ameliorated NASH in a CDA diet-induced murine model. The therapeutic effects of Alisol B (100 mg/kg, once daily, p.o.) were evaluated in CDA diet-fed mice as described in the Materials and Methods. (**A**) Body weight and (**B**) average food intake were measured. (**C**) H&E staining (200× magnification) and Sirius Red staining (100× magnification) were performed. (**D**) NAS score, steatosis score, ballooning score, and inflammation score were quantified. (**E**) Serum AST and (**F**) ALT levels, (**G**) liver TG, and (**H**) hepatic mRNA levels of inflammatory cytokines were measured. (**I**) Percentage of collagen deposition was calculated. (**J**) Western blot analysis of hepatic α-SMA and Col-1a1 was conducted and quantified. Data are expressed as mean ± SD, *n* = 8. # *p* < 0.05, ## *p* < 0.01 compared with the normal control group; * *p* < 0.05, ** *p* < 0.01 compared with the CDA group.

The therapeutic effects of Alisol B against NASH were further validated in a CDA diet-induced murine model. As shown in Figure 2A,B, Alisol B did not have significant effects on body weight and average food intake. H&E staining revealed that Alisol B treatment caused an obvious alleviation on hepatic steatosis (Figure 2C), in agreement with the decrease on NAS and steatosis score (Figure 2D). The serum AST level of CDA-diet-induced NASH mice was significantly decreased by 29.2% (Figure 2E), while the serum ALT level was not significantly affected by Alisol B (Figure 2F). The hepatic TG content was decreased by 28.1% after chronic treatment with Alisol B (Figure 2G). Moreover, the mRNA levels of inflammatory cytokines such as TNF-α, TGF-β, and IL-6 were suppressed by Alisol B as well (Figure 2H). The full scan of Sirius Red staining of liver sections indicated that Alisol B treatment exhibited a remarkable improvement on collagen deposition (Figure 2I). Accordingly, the elevated expression of α-SMA and Col-1a1 induced by CDA diet was significantly suppressed by the treatment of Alisol B (Figure 2J).

### 3.3. RNA-Seq Analysis

**Figure 3:**
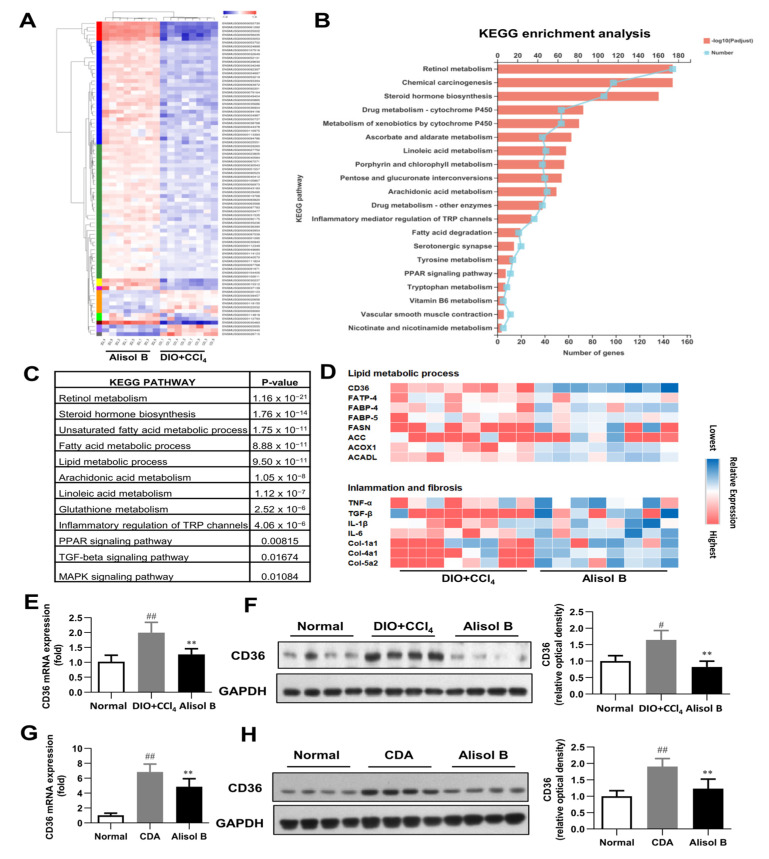
RNA-seq analysis. (**A**) Heat map, (**B**) KEGG enrichment analysis, (**C**) enriched KEGG biological pathway, and (**D**) kay genes expression involved in lipid metabolic process, inflammation, and fibrosis were performed in the liver of DIO+CCl_4_-induced mice. (**E**) RT-PCR analysis and (**F**) Western blot analysis of CD36 expression were conducted in DIO+CCl_4_-induced mice. (**G**) RT-PCR analysis and (**H**) Western blot analysis of CD36 expression were conducted in CDA-diet-fed mice. Data are expressed as mean ± SD, *n* = 8. # *p* < 0.05, ## *p* < 0.01 compared with the normal control group; ** *p* < 0.01 compared with the DIO+CCl_4_ group or CDA group.

To investigate the possible mechanisms involved in the therapeutic effects of Alisol B on NASH, RNA-seq analysis was performed with liver tissues of a DIO+CCl_4_-induced murine NASH model. As shown in Figure 3A, 26,394 different transcripts were identified, among which 302 genes were significantly upregulated and 396 genes were downregulated (fold change greater than 2 and *p*-value less than 0.01). KEGG pathway analysis determined retinol metabolism as the top upregulated pathway by Alisol B treatment (Figure 3B). Moreover, the unsaturated fatty acid metabolic process, inflammatory mediator regulation, and PPAR and MAPK signaling were also strongly affected by Alisol B (Figure 3C). As shown in Figure 3D, the representative genes in the lipid metabolic processes, inflammation, and fibrosis were remarkably regulated by Alisol B. CD36, a member of the scavenger receptor family closely related to FFA uptake and immunological regulation, was found to be markedly downregulated by Alisol B, whereas other genes involved in FFA uptake such as FABP-4, FABP-5, and FATP-4 were not significantly affected. The downregulation of CD36 was further confirmed by RT-PCR analysis in that the hepatic gene expression of CD36 was significantly decreased by 36.4% and 29.1% upon Alisol B treatment in DIO+CCl_4_ and CDA-diet-induced NASH mice, respectively (Figure 3E,G). The decreased protein level of CD36 was observed in both murine NASH models as well (Figure 3F,H).

### 3.4. Alisol B Ameliorated Cellular TG Accumulation in Primary Hepatocytes by Inhibiting FFA Uptake in a CD36-Dependent Manner

**Figure 4:**
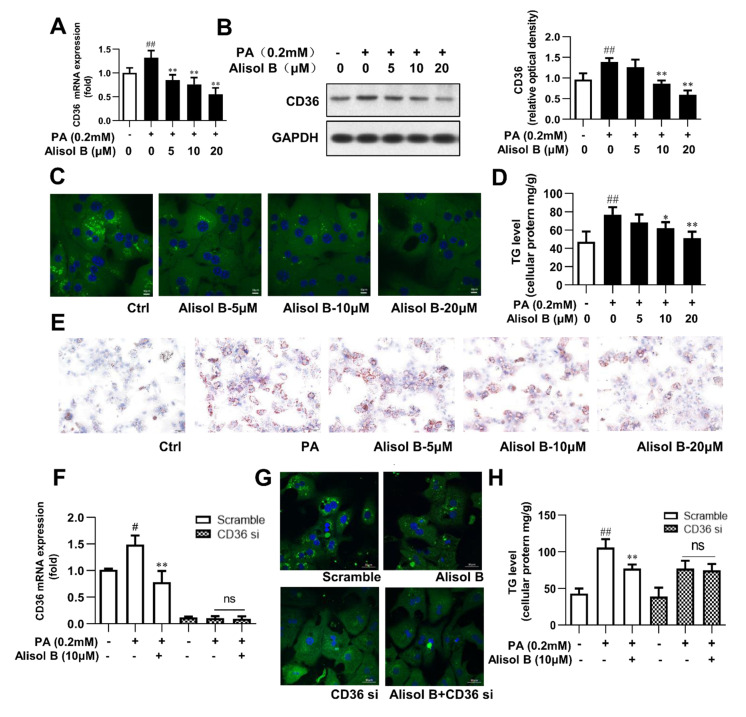
Alisol B ameliorated cellular TG accumulation in primary hepatocytes by inhibiting FFA uptake in a CD36-dependent manner. Mouse primary hepatocytes were incubated with indicated concentration of Alisol B under PA (0.2 mM)-stimulated condition. (**A**) RT-PCR analysis and (**B**) Western blot analysis of CD36 expression, (**C**) BODIPY-C16 fluorescence (600× magnification), (**D**) cellular TG, and (**E**) Oil Red O staining (200× magnification) were conducted. (**F**–**H**) Primary hepatocytes were pretreated with CD36 siRNA for 24 h and then treated with Alisol B (10 μM) under PA-induced conditions. (**F**) CD36 mRNA level, (**G**) BODIPY-C16 fluorescence (600× magnification), and (**H**) cellular TG were examined. Data are expressed as mean ± SD, *n* = 5. # *p* < 0.05, ## *p* < 0.01 compared with the normal control group; * *p* < 0.05, ** *p* < 0.01 compared with the PA-induced group.

The downregulation of CD36 by Alisol B was further studied in mouse primary hepatocytes. As shown in Figure 4A,B, PA significantly increased the mRNA and protein levels of CD36, and this effect was dose-dependently suppressed by Alisol B. Immunofluorescence staining indicated that fatty acid uptake was markedly inhibited by Alisol B (Figure 4C), and the PA-induced intracellular TG accumulation was significantly decreased by 20.0% and 33.5%, with the treatment of 10 and 20 μM Alisol B, respectively (Figure 4D). Accordingly, Oil Red O staining showed that the accumulation of lipid droplets induced by PA could be clearly reduced by the treatment of Alisol B in mouse primary hepatocytes (Figure 4E). To analyze the role of CD36 in Alisol B-inhibited fatty acid uptake and TG accumulation, CD36 knockdown using siRNA was performed in mouse primary hepatocytes. After siRNA interference, the CD36 gene level was decreased by 90.1% compared with the negative control group (Figure 4F). Fatty acid uptake was significantly suppressed after CD36 knockdown, and the inhibition of Alisol B on FFA uptake was fully abolished (Figure 4G). Similarly, the cellular TG content induced by PA was significantly reduced in CD36-siRNA-transfected hepatocytes, and Alisol B’s improving effect on cellular TG accumulation was deprived by CD36 siRNA interference (Figure 4H). These results suggested that Alisol B suppressed cellular lipid accumulation in primary hepatocytes via inhibiting FFA uptake in a CD36-dependent manner.

### 3.5. Alisol B Inhibited Oxidative Stress and Inflammation in Primary Hepatocytes in a CD36-Dependent Manner

**Figure 5:**
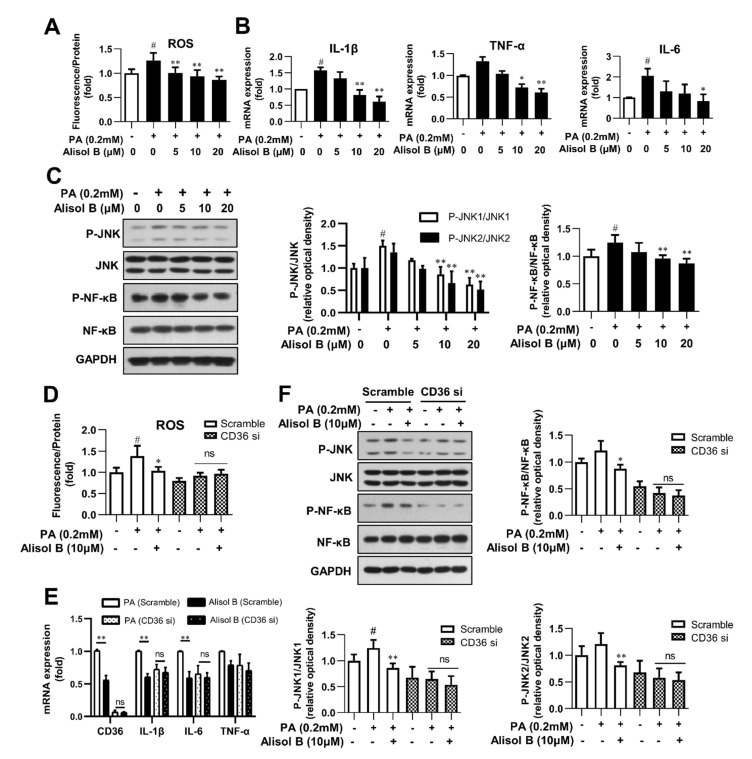
Alisol B inhibited oxidative stress and inflammation in primary hepatocytes in a CD36-dependent manner. Mouse primary hepatocytes were incubated with indicated concentration of Alisol B under PA (0.2 mM)-stimulated conditions. (**A**) Cellular ROS level; (**B**) the mRNA levels of IL-1β, IL-6, and TNF-α; and (**C**) JNK1/2 and NF-κB phosphorylation were detected. (**D**–**F**) Primary hepatocytes were pretreated with CD36 siRNA for 24 h and then treated with Alisol B (10 μM) under PA-induced conditions. (**D**) Cellular ROS; (**E**) the mRNA levels of IL-1β, IL-6, and TNF-α; and (**F**) JNK1/2 and NF-κB phosphorylation were detected. Data are expressed as mean ± SD, *n* = 5 for all of the groups, except for *n* = 6 in Figure (**F**). # *p* < 0.05 compared with the normal control group; * *p* < 0.05, ** *p* < 0.01 compared with the PA-induced group.

Excess lipid accumulation is closely associated with elevated oxidative stress and chronic inflammation [27]. Considering the indispensable role of CD36 on Alisol B-inhibited fatty acid uptake and lipid accumulation, we further explored the effects of Alisol B and the involvement of CD36 on ROS, inflammatory cytokines, and related signaling in mouse primary hepatocytes. As shown in Figure 5A, 10 and 20 μM Alisol B decreased the intracellular ROS level by 25.9% and 31.8%, respectively. Meanwhile, the mRNA levels of TNF-α, IL-1β, and IL-6 were markedly decreased by Alisol B (Figure 5B). In addition, the phosphorylation of JNK1/2 and NF-κB, the critical mediators of oxidative stress and inflammation, were also significantly suppressed by Alisol B in mouse primary hepatocytes (Figure 5C). Then, CD36 siRNA was employed to address whether CD36 was necessary in Alisol B-inhibited oxidative stress and inflammation. As shown in Figure 5D,E, the cellular ROS generation and mRNA levels of inflammatory cytokines were obviously decreased in CD36-siRNA-transfected hepatocytes. Moreover, CD36 siRNA interference deprived Alisol B of its capability in suppressing ROS generation and mRNA levels of inflammatory cytokines in mouse primary hepatocytes. Meanwhile, the reduced phosphorylation of JNK1/2 and NF-κB caused by Alisol B were also fully abrogated after CD36 knockdown in mouse primary hepatocytes (Figure 5F). These data implied that Alisol B attenuated PA-induced oxidative stress and inflammation in mouse primary hepatocytes by downregulating CD36 expression and inhibiting the JNK/NF-κB signaling pathway.

### 3.6. Alisol B Decreased CD36 Expression through Downregulating PPARγ, and This Effect Was Independent of FXR

**Figure 6:**
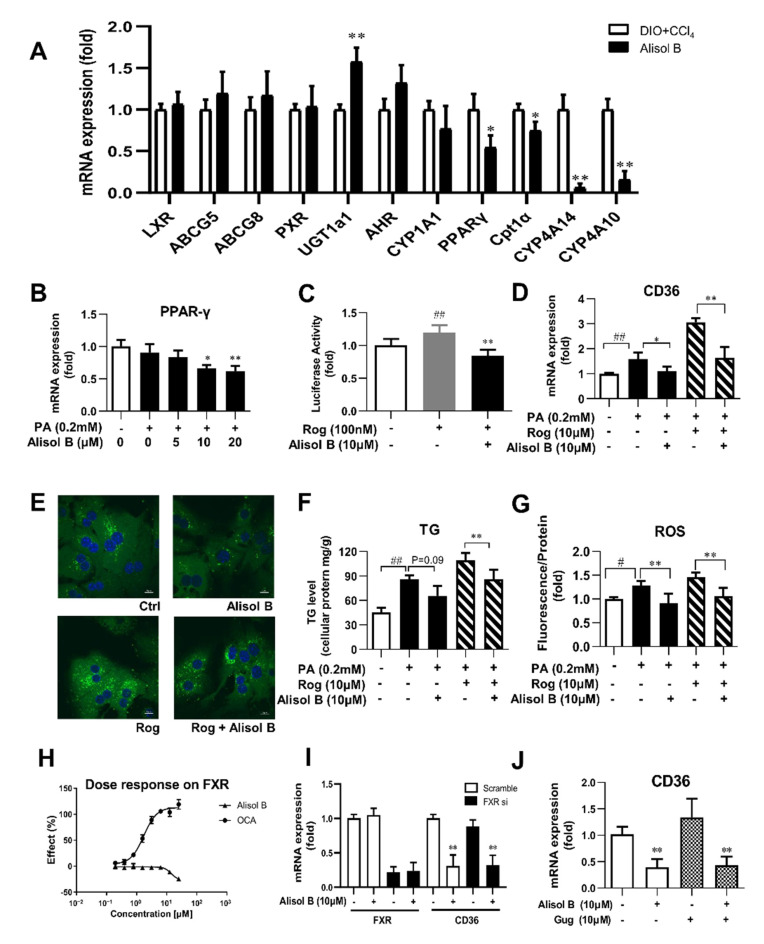
Alisol B suppressed CD36 expression through downregulating PPARγ, and this effect was independent of FXR. (**A**) The mRNA expression of hepatic LXR, PXR, AHR, PPARγ, and their target genes were examined in DIO+CCl_4_-induced NASH mice. (**B**) The gene expression of PPARγ was measured in PA-induced primary hepatocytes treated with Alisol B. (**C**) Huh7 cells were transiently transfected and then treated with Alisol B (10 μM) in the presence of Rosiglitazone. The transactivation activity of PPARγ was detected in luciferase reporter assay. (**D**–**G**) Mouse primary hepatocytes were treated with Alisol B (10 μM) and Rosiglitazone (10 μM) under PA-stimulated conditions. (**D**) CD36 mRNA level, € BODIPY-C16 fluorescence (600× magnification), (**F**) cellular TG, and (**G**) cellular ROS were evaluated. (**H**) Huh7 cells were transiently transfected and then treated with Alisol B. The agonistic activity on FXR was detected in luciferase reporter assay. (**I**) The mRNA levels of FXR and CD36 in FXR knockdown hepatocytes treated with Alisol B (10 μM) were detected. (**J**) The mRNA level of CD36 in hepatocytes treated with Alisol B (10 μM) in the presence of Gugglusterone (10 μM) was detected. Data in (**A**) are expressed as mean ± SD, *n* = 8. * *p* < 0.05, ** *p* < 0.01 compared with DIO+CCl_4_ group. Data in (**B**–**H**) are expressed as mean ± SD, *n* = 5. # *p* < 0.05, ## *p* < 0.01 compared with normal control group; * *p* < 0.05, ** *p* < 0.01 compared with the PA-induced group or Rosiglitazone-induced group. Data in (I,J) are expressed as mean ± SD, *n* = 5. ** *p* < 0.01 compared with the normal control group.

Since the transcription of CD36 is regulated by several nuclear receptors such as LXR, PXR, PPARγ, and AHR, the expression of these nuclear receptors and their target genes were investigated in the liver of DIO+CCl_4_-induced NASH mice. As shown in Figure 6A, the hepatic mRNA expression of PPARγ and its downstream target genes were markedly reduced by the treatment of Alisol B, whereas no significant decrease was observed in the expression of LXR, AHR, PXR, and their target genes. Consistently, the gene expression of PPARγ was also suppressed by Alisol B in mouse primary hepatocytes (Figure 6B). Meanwhile, Alisol B antagonized the activation of PPARγ stimulated by Rosiglitazone, a well-known PPARγ agonist, in transactivation assay (Figure 6C). Moreover, Alisol B significantly suppressed the elevated CD36 gene expression induced by Rosiglitazone in mouse primary hepatocytes (Figure 6D). The increased fatty acid uptake, cellular TG content, and ROS generation induced by Rosiglitazone were also alleviated by the treatment of Alisol B (Figure 6E–G). Considering the early report that Alisol B’s analogue protected against NAFLD via FXR activation [25], FXR luciferase reporter assay was carried out, while Alisol B showed no direct agonistic activity on FXR (Figure 6H). Moreover, FXR siRNA and FXR antagonist Gugglusterone were employed to further address whether FXR was necessary on Alisol B-inhibited CD36 expression. As shown in Figure 6I,J, 10 μM Alisol B significantly decreased the mRNA level of CD36, and this suppression was not affected by either FXR siRNA or FXR antagonist. These results indicated that Alisol B suppressed CD36 expression via downregulating PPARγ.

### 3.7. Alisol B Decreased CD36 Expression and Attenuated Hepatocyte Lipid Accumulation and Lipotoxicity via RARα-HNF4α-PPARγ Transcriptional Cascade

**Figure 7:**
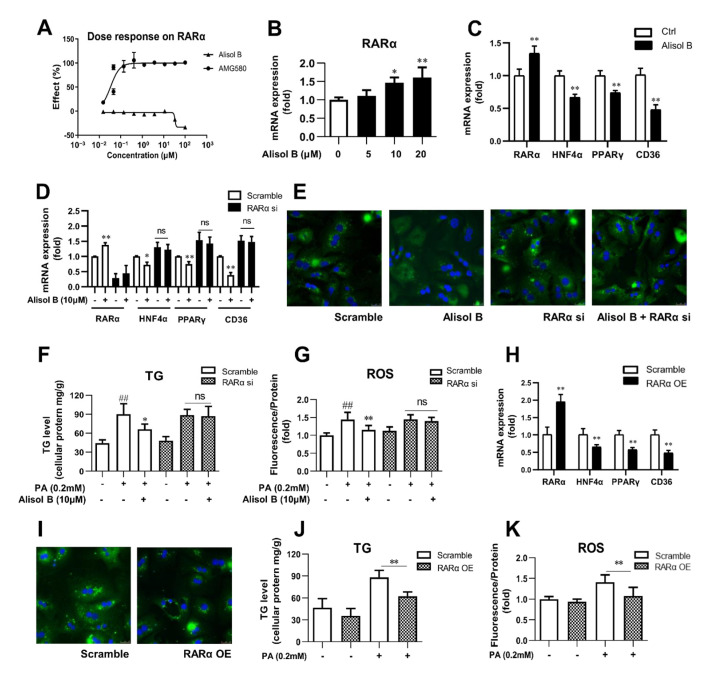
Alisol B decreased CD36 expression and attenuated hepatocyte lipid accumulation and lipotoxicity via regulating RARα-HNF4α-PPARγ cascade. (**A**) HEK293 cells were transient transfected and then treated with Alisol B. The agonistic activity on RARα was detected in luciferase reporter assay. (**B**) The gene expression of RARα was detected. (**C**) The mRNA levels of RARα, HNF4α, PPARγ, and CD36 were measured in hepatocytes treated with Alisol B (10 μM). (**D**–**G**) Primary hepatocytes were transfected with RARα siRNA for 24 h and then treated with Alisol B (10 μM). (**D**) The mRNA levels of RARα, HNF4α, PPARγ, and CD36; (**E**) BODIPY-C16 fluorescence (600× magnification); (**F**) cellular TG; and (**G**) cellular ROS were detected. (**H**–**K**) Primary hepatocytes were overexpressed with RARα via transient plasmid transfection for 24 h. (**H**) The mRNA levels of RARα, HNF4α, PPARγ, and CD36; (**I**) BODIPY-C16 fluorescence (600× magnification); (**J**) cellular TG; and (**K**) cellular ROS were conducted. Data are expressed as mean ± SD, *n* = 5. In (**F**,**G**,**J**,**K**), ## *p* < 0.01 compared with normal control group; * *p* < 0.05, ** *p* < 0.01 compared with the PA-induced group. In the other figures, * *p* < 0.05, ** *p* < 0.01 compared with the normal control group.

RARα overexpression was reported to alleviate hepatic lipid accumulation via RARα-regulated PPARγ cascade [19]. To find out the molecular mechanism by which Alisol B suppressed PPARγ expression and whether CD36 is involved in RARα’s regulation on hepatic lipid metabolism, RARα luciferase reporter assay was firstly performed, and Alisol B showed no direct agonistic activity on RARα (Figure 7A). However, Alisol B dose-dependently increased the mRNA level of RARα in mouse primary hepatocytes (Figure 7B). The RARα-mediated transcriptional cascades were further evaluated, and as shown in Figure 7C, 10 μM Alisol B significantly increased RARα expression and decreased CD36 expression, along with the downregulation of HNF4α and PPARγ. More importantly, the Alisol B-inhibited CD36 was fully abrogated after RARα knockdown in primary hepatocytes (Figure 7D). Meanwhile, the suppressed FFA uptake, attenuated TG accumulation, and ROS generation caused by Alisol B were completely abolished by RARα interference in mouse primary hepatocytes (Figure 7E–G). Then, to better understand the role of RARα on the regulation of CD36, RARα overexpression was conducted in mouse primary hepatocytes. As shown in Figure 7H, the mRNA level of RARα was effectively increased by 2.0-fold compared with vector control, and a profound suppression on CD36 was observed in RARα-overexpressed hepatocytes, accompanied with the downregulation of HNF4α and PPARγ. Accordingly, FFA uptake was suppressed, and cellular TG accumulation and ROS generation were subsequently reduced after RARα overexpression in mouse primary hepatocytes (Figure 7I–K). Taken together, these results suggested the indispensable role of RARα-HNF4α-PPARγ transcriptional cascade on Alisol B-suppressed CD36 expression and valued the important role of RARα-PPARγ-CD36 cascade on the regulation of lipid accumulation and oxidative stress in hepatocytes.

### 3.8. Chronic Treatment of Alisol B Regulated RARα-PPARγ-CD36 Transcriptional Cascade and Inhibited JNK/NF-κB Signaling Pathway in NASH Mice

**Figure 8:**
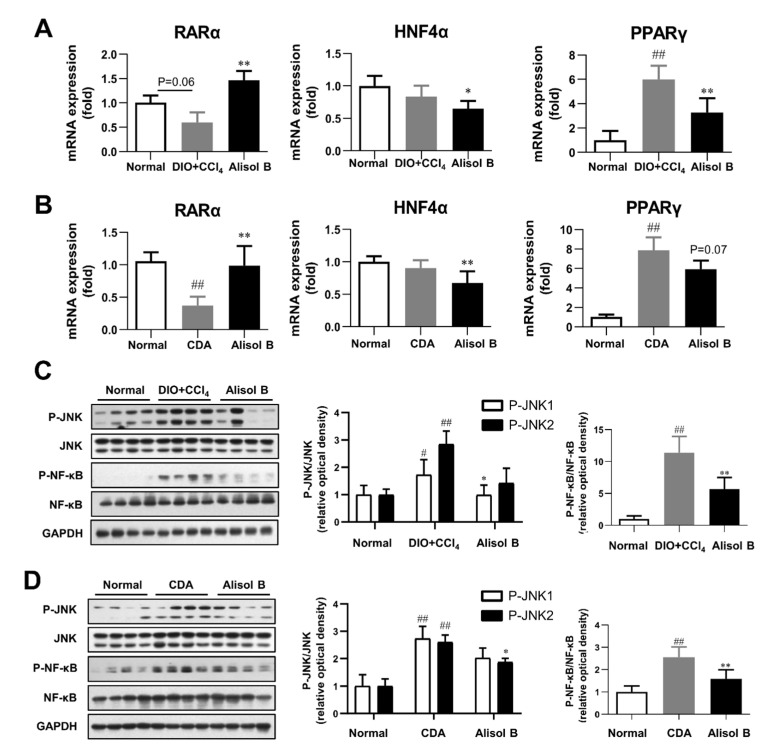
Alisol B regulated RARα-mediated transcriptional cascade and inhibited JNK/NF-κB signaling pathway in NASH mice. Hepatic mRNA levels of RARα, HNF4α, and PPARγ were examined in (**A**) DIO+CCl_4_-induced NASH mice and (**B**) CDA diet-induced NASH mice. JNK1/2 and NF-κB phosphorylation were detected in (**C**) DIO+CCl_4_-induced NASH mice and (**D**) CDA-diet-induced NASH mice. Data are expressed as mean ± SD, *n* = 8. # *p* < 0.05, ## *p* < 0.01 compared with the normal control group; * *p* < 0.05, ** *p* < 0.01 compared with the DIO+CCl_4_ group or CDA group.

The effects of chronic administration of Alisol B on hepatic RARα-PPARγ-CD36 transcriptional cascade and JNK/NF-κB signaling pathway were investigated in vivo. As shown in Figure 8A,B, the gene expression of RARα was markedly decreased in both DIO+CCl_4_ and CDA diet-induced NASH mice compared with normal control mice, which was profoundly increased by 2.5-fold and 2.7-fold after the chronic treatment of Alisol B, respectively. Moreover, the mRNA levels of HNF4α and PPARγ were markedly suppressed by Alisol B in both murine NASH models. Furthermore, hepatic JNK/NF-κB signaling pathway was detected, and the phosphorylation of JNK1/2 and NF-κB in DIO+CCl_4_ and CDA diet-induced mice were obviously increased in comparison with that of normal control mice, which were significantly suppressed by Alisol B (Figure 8C,D). Therefore, the in vivo data are highly concordant with the in vitro outcomes and support that Alisol B regulated RARα-PPARγ-CD36 cascade and further alleviated the phenotypes of NASH.

## 4. Discussion

Although the global prevalence of NASH is increasing, efficacious drugs are still lacking [33]. Discovering lead compounds from herb medicine might be a valuable drug discovery strategy for the treatment of NASH. In the present study, we found Alisol B, a compound isolated from *Alisma orientalis* (*Sam.*), showed significant therapeutic effects on DIO+CCl_4_ and CDA diet-induced murine NASH models. More importantly, we identified the novel mechanisms that Alisol B attenuated hepatocyte lipid accumulation and lipotoxicity via regulating RARα-PPARγ-CD36 transcriptional cascade, thereby ameliorating the development of NASH.

*Alisma orientalis* (*Sam.*) is a traditional medicinal plant with bioactive effects on metabolic syndrome [21,22]. The anti-NAFLD activity of a few active constituents of *Alisma orientalis* (*Sam.*), such as Alisol A 24-acetate [34] and Alisol B 23-acetate [27], have been studied. However, Alisol B, one of the most abundant components in *Alisma orientalis* (*Sam.*), was only reported to have potential ability on anti-hyperlipidemia [28], and its therapeutic effects against NASH and the specific mechanisms remain unclear. The DIO+CCl_4_-induced murine model [35] and CDA-diet-induced murine model [36] are both well-established experimental NASH models with some features similar to the pathological features of human NASH. Here, we evaluated the effects of Alisol B in both models, and the results showed that Alisol B significantly attenuated hepatic steatosis and decreased inflammatory cytokine expression and lipid peroxidation, accompanied with the attenuation of fibrosis. These results indicate the potential therapeutic efficacy of Alisol B for the treatment of NASH.

Considering the unclear mechanisms and unknown intracellular target of Alisol B, RNA-seq analysis was conducted, and the significant suppressed CD36 expression and obvious changed retinol metabolism pathway were discovered in the liver of DIO+CCl_4_-induced NASH mice. Mounting evidence have revealed that hepatic lipid accumulation results from an imbalance between lipid acquisition and lipid disposal. As a major way to acquire lipid from the circulatory system, FFA uptake is largely regulated by fatty acid translocase CD36 [37] and fatty-acid-binding proteins located on the hepatocyte plasma membrane. Here, we found that Alisol B remarkably inhibited lipid accumulation in PA-induced mouse primary hepatocytes, along with an obvious suppression on FFA uptake. Moreover, the gene and protein levels of CD36 were markedly suppressed by Alisol B, while other FFA transporters such as FATP-4, FABP-4, and FABP-5 were not significantly affected. Importantly, Alisol-B-reduced cellular FFA uptake and lipid accumulation could be completely abolished after CD36 knockdown, indicating that Alisol B suppressed cellular lipid accumulation via inhibiting FFA uptake in a CD36-dependent manner.

Lipid overload of hepatocytes is of central importance during NAFLD procession, since lipid peroxidation and toxic lipid species lead to cytokine release and pro-inflammatory signaling activation along with activation of hepatic stellate cells and immune cells [2]. As a multifunctional receptor with a special extracellular domain, CD36 has been reported to recognize pathogen-related molecular patterns with the consequent activation of inflammatory cascades in immunocytes [38]. However, the modulation of CD36 on inflammatory responses and oxidative stress in hepatocytes is still unclear. Here, Alisol B was found to relieve cellular ROS level and decrease inflammatory cytokines expression in mouse primary hepatocytes, along with a robust blockade of JNK/NF-κB pathway. Importantly, all these improving effects of Alisol B were dependent on CD36 suppression. Our results showed the indispensable role of CD36 on Alisol-B-inhibited oxidative stress and inflammatory responses in hepatocytes. Moreover, we revealed that CD36 might represent a crucial link between excess lipid accumulation and lipotoxicity, which synergistically contributed to the development of NASH.

Early studies have reported that CD36 is a common target of several nuclear receptors such as PPARs, LXR, PXR, and AHR [19]. Mounting evidence has revealed the critical role of nuclear receptors in the pathogenesis and treatment of NAFLD [39]. However, whether the biological actions of protostane-type triterpenoids in *Alisma orientalis* (*Sam.*) are related to nuclear receptors still remains under debate, with some studies reporting that Alisol B 23-acetate showed FXR agonistic activity [25,27], while another study argued that no ligand-binding activity of FXR was found [26]. Considering these contradictory results, we carried out luciferase reporter assay and confirmed that Alisol B did not have the direct agonistic activity on FXR. Moreover, we demonstrated that Alisol B’s suppression on CD36 was not related to FXR since this effect was not affected by either FXR knockdown or cotreated with FXR antagonist. Then, the reported nuclear receptors regulating CD36 transcription were detected in a DIO+CCl_4_-induced murine model, and PPARγ was found markedly reduced after the chronic administration of Alisol B, with no significant decrease observed on LXR, AHR, PXR, and their target genes. Similar to the in vivo result, Alisol B obviously reduced PPARγ expression in mouse primary hepatocytes and suppressed PPARγ transactivation activity induced by PPARγ agonist Rosiglitazone, suggesting that Alisol B decreasing CD36 expression might have been due to the downregulation on PPARγ.

An early study has identified that RARα overexpression alleviated hepatic steatosis via suppressing HNF4α-regulated PPARγ expression [19], but whether CD36 is involved in RARα’s regulation on hepatic lipid metabolism is unknown. Meanwhile, the RNA-seq analysis showed that retinol metabolism was the top regulated pathway induced by Alisol B in DIO+CCl_4_-induced NASH mice, which prompted us to pay more attention to the relationship between RARα and CD36 expression. Our study firstly revealed that Alisol B significantly increased RARα gene expression with no direct RARα agonistic activity, and markedly decreased CD36 expression, along with the suppression on HNF4α and PPARγ in mouse primary hepatocytes. More importantly, Alisol B-suppressed CD36 expression could be fully blocked by RARα knockdown. Consequently, the reduced FFA uptake, attenuated TG accumulation, and ROS generation caused by Alisol B were completely abolished by RARα interference as well, indicating that Alisol B decreased CD36 expression via regulating RARα-HNF4α-PPARγ transcriptional cascade. The important role of RARα on the regulation of CD36 was further confirmed by conducting RARα overexpression in mouse primary hepatocytes. As expected, RARα overexpression significantly decreased the mRNA level of CD36, accompanied with the suppression on HNF4α and PPARγ. Accordingly, FFA uptake, cellular TG accumulation, and ROS generation were obviously suppressed, which further strengthened the important role of RARα-HNF4α-PPARγ transcriptional cascade on CD36 expression and regulation of lipid accumulation and lipotoxicity in hepatocytes.

The regulation of Alisol B on RARα-PPARγ-CD36 transcriptional cascade and related signaling were evaluated in vivo. Interestingly, the gene level of RARα was found to be remarkably suppressed in both murine NASH models. Similar with the results in mouse primary hepatocytes, chronic administration of Alisol B significantly upregulated the impaired mRNA level of RARα, decreased CD36 expression along with the down-regulation of HNF4α and PPARγ, and further suppressed phosphorylation of JNK1/2 and NF-κB in both DIO+CCl_4_ and CDA-diet-induced NASH mice. Additionally, chronic administration of Alisol B alleviated hepatic steatosis, along with the decreased inflammatory cytokine expression and improved lipid peroxidation in both murine NASH models, which were highly concordant with the in vitro improvements on hepatocyte lipid accumulation and lipotoxicity. Some studies have implicated the diverse mechanisms of *Alisma orientalis* extracts against NASH, and Alisol A 24-acetate and Alisol B 23-acetate were reported to ameliorate NASH via stimulating autophagy and regulating AMPK/mTOR signaling [34] or activating FXR [27], respectively. Differently, our study uncovered the novel molecular mechanisms of Alisol B on hepatic steatosis and lipotoxicity via regulating RARα-PPARγ-CD36 transcriptional cascade. To date, atRA [16] and synthetic RAR agonists [40] have been reported to alleviate NAFLD in preclinical studies; however, little research has focused on regulating RARα expression and RARα-mediated transcriptional cascade for the treatment of NASH. Although the precise mechanisms about how Alisol-B-upregulated RARα expression still need to be further investigated, our study did highlight the important role of RARα on hepatic steatosis, oxidative stress, and inflammation, which generated a novel therapeutic strategy targeting RARα-PPARγ-CD36 cascade for the treatment of NASH.

## 5. Conclusions

In conclusion, this is the first report that Alisol B alleviated hepatocyte lipid accumulation and lipotoxicity via regulating RARα-PPARγ-CD36 cascade, thereby attenuating NASH in mice. We discovered the novel mechanism that Alisol B upregulated the disrupted expression of RARα and then decreased CD36 expression via RARα-HNF4α-PPARγ transcriptional cascade, which further alleviated hepatic steatosis, oxidative stress, inflammation, and fibrosis in the liver of NASH mice. These findings uncovered the underlying mechanism of Alisol B via regulating RARα-PPARγ-CD36 cascade to alleviate NASH and highlighted Alisol B’s promising potential for the treatment of NASH.

## Figures and Tables

**Table 1:** Primer sequences used for RT-PCR analysis.

Primer	Sequence (5′-3′)
ABCG5-F	ATCCAACACCTCTATGCTAAATCAC
ABCG5-R	TACATTATTGGACCAGTTCAGTCAC
ABCG8-F	GAGAGCTTCACAGCCCACAA
ABCG8-R	GCCTGAAGATGTCAGAGCGA
AHR-F	TTCTTAGGCTCAGCGTCAGCTA
AHR-R	GCAAATCCTGCCAGTCTCTGAT
CD36-F	ATGGGCTGTGATCGGAACTG
CD36-R	GTCTTCCCAATAAGCATGTCTCC
Col-1a1-F	ACCTGTGTGTTCCCTACTCA
Col-1a1-R	GACTGTTGCCTTCGCCTCTG
CPT1α-F	TGGCATCATCACTGGTGTGTT
CPT1α-R	GTCTAGGGTCCGATTGATCTTTG
CYP1A1-F	CCTCATGTACCTGGTAACCA
CYP1A1-R	AAGGATGAATGCCGGAAGGT
CYP4A10-F	TCCAGCAGTTCCCATCACCT
CYP4A10-R	TTGCTTCCCCAGAACCATCT
CYP4A14-F	CCCAAAGGTATCACAGCCACAA
CYP4A14-R	CAGCAATTCAAAGCGGAGCAG
GAPDH-F	GGATTTGGCCGTATTGGGCG
GAPDH-R	CAGTAGAGGCAGGGATGATG
HNF4α-F	GGTTTAGCCGACAATGTGTGG
HNF4α-R	TCCCGCTCATTTTGGACAGC
IL-1β-F	ACCCTGCAGCTGGAGAGTGT
IL-1β-R	TTGACTTCTATCTTGTTGAAGACAAACC
IL-6-F	CCACTTCACAAGTCGGAGGCTTA
IL-6-R	GCAAGTGCATCATCGTTGTTCATAC
LXR-F	TCTGGAGACATCTCGGAGGTA
LXR-R	GGCTCACCAGTTTCATTAGCA
PPAR-γ-F	GGGGCCTGGACCTCTGCTGGGGATCT
PPAR-γ-R	GGCCAGAATGGCATCTCTGTGTCAA
PXR-F	CCCATCAACGTAGAGGAGGA
PXR-R	GGGGGTTGGTAGTTCCAGAT
RARα-F	GGCATCAACAAGCAAGAGTTTGGC
RARα-R	CTTTTTGGTGAGGTGATCTGTCCC
TGF-β-F	GTGTGGAGCAACATGTGGAACTCTA
TGF-β-R	TTGGTTCAGCCACTGCCGTA
TNF-α-F	TATGGCCCAGACCCTCACA
TNF-α-R	GGAGTAGACAAGGTACAACCCATC
UGT1a1-F	AGATTACCCCAGGCCCATC
UGT1a1-R	ATGGCTTTCTTCTCCGGAAT
α-SMA-F	GACGCTGAAGTATCCGATAGAACACG
α-SMA-R	CACCATCTCCAGAGTCCAGCACAAT

## Data Availability

The data presented in this study are available on request from the corresponding author.
